# A survey of cricoid pressure application in a single institution in Ethiopia

**DOI:** 10.1186/s13104-019-4586-4

**Published:** 2019-08-28

**Authors:** Naod Bulti Etanaa, Kore Menjie Benwu

**Affiliations:** Mekelle University, Mekelle, Tigray Region Ethiopia

**Keywords:** Cricoid pressure, Anesthetists, Practice, Knowledge, Rapid sequence induction

## Abstract

**Objective:**

The aim of this survey is to determine the standard of practice of cricoid pressure application on rapid sequence induction in Ayder comprehensive specialized hospital from April 3 to May 3, 2019.

**Results:**

A total of 30 anesthetists were involved in the study with a response rate of 87%. Ninety percent of the respondents do not mask ventilate during rapid sequence induction and they do aspirate the naso-gastric tube if present. Almost half of the respondents have witnessed regurgitation during application of cricoid pressure and 93% do not remove the naso-gastric tube before rapid sequence induction. Seventy percent had experienced difficulty of endotracheal intubation during application of cricoid pressure. All of the respondents had less than 10 years of working experience as anesthetist.

## Introduction

Cricoid pressure was initially described by Sellick as a simple method to protect patients from regurgitation of gastric contents during the time of endotracheal intubation [1]. To practice safe and effective use of this maneuver it requires training and knowledge of the related anatomy, technique of application of cricoid pressure and good information about its associated complication.

As originally described by Selilick, immediately before intravenous induction of anesthesia, while the patient is still awake cricoid pressure is applied lightly (20 N or 2 kg) by trained assistant. Once the patient has lost consciousness, the force on the cricoid cartilage is increased to (40 N or 4 kg). Cricoid pressure is released after tracheal intubation is confirmed [2].

Vanner initially staged the amount of force to be applied, based on a cadaver study. He advocated an initial force of 20 N for awake patients and 30 N after the loss of consciousness in subjects. Subsequently in 1999 he suggested that the initial force be decreased to 10 N in awake subjects and then slowly increased to 30 N as the patient lost consciousness [3].

Despite the fact that cricoid pressure has become widely accepted as a standard of practice during anesthesia in the United Kingdom (UK) and United states of America (USA) and many parts of the world, none of the papers confirm the perceived clinical benefits of cricoid pressure in reducing the incidence of aspiration during an emergency rapid sequence induction [4].

Though there continues to be controversy regarding the efficacy of cricoid pressure and its safety, it is still a standard practice of most anesthesiologists. Various studies assessing knowledge e of practitioners regarding cricoid pressure (CP) and the effect of training on them have been done. The uniform conclusion in all these studies was that theoretical knowledge of CP was poor in all categories of tested people including anesthesiologists. These studies included anesthesiologists in Sweden [5] and anesthetic assistants in the UK. Neither of these studies documented whether cricoid pressure had been taught to them as an independent skill or not.

The aim of this institutional survey is to determine the standard of practice of cricoid pressure application for rapid sequence induction in Ayder comprehensive specialized hospital (ACSH) and forward recommendations based on observed performances.

## Main text

### Method and materials

This study will be conducted in Ayder comprehensive specialized hospital in Mekelle which is found in southern part of Tigray, Ethiopia. The hospitals’ anesthetic service is provided by 34 non-physician anesthetists: of whom 12 had Master of sciences (MSc) in anesthesia and 22 had Bachelor of Science (BSc) in anesthesia. It provides more than 7000 surgical and anesthetic care using 9 operating rooms for 10 specialties and subspecialties including neurosurgery, obstetric surgery and pediatric surgery.

This cross sectional, institutional survey was conducted by a means of self-administered structured questionnaires. After taking oral and written informed consent, volunteers were asked to fill up the questionnaires about their routine practice and knowledge regarding cricoid pressure application. The results were analyzed using SPSS (version 20) after the data was compiled and cleansed. Descriptive statistics result was presented as percentages from total responses. The study was conducted from April 3 to May 3, 2019 at Ayder comprehensive specialized hospital.

### Results

A total of 30 anesthetists were involved in the study with a response rate of 87%. From this, 83% had applied cricoid pressure for rapid sequence induction more than 50 cases in their working experience’s. Most, 93%, had learned how to apply cricoid pressure on a real patient during their clinical practice from anesthesia instructors and 7% have learned by reading only (Table 1).

**Table 1:** Characteristic of anesthetists working in Ayder comprehensive specialized hospital April 3–May 3, 2019

Characteristics of participants	Number of anesthetists	Percentage
Year of experience (years)		
< 2	13	43.3
2–5	10	33.3
5–10	7	23.3
> 10	0	0
Have you ever applied cricoid pressure		
Yes	30	100
No	–	–
Number of cricoid pressure application		
< 50 times	4	13.4
> 50 times	26	86.6
How did you learn to apply cricoid pressure		
Shown on a patient during clinical practice or student attachment	28	93
By reading only	2	6.7
By practicing on a model or manikin	–	–
I have never been taught about it	–	–

We also assessed knowledge of cricoid application. The correct anatomic position of cricoid cartilage was identified by 83%. Hundred percent of the participants replied cricoid pressure is applied to prevent aspiration of stomach contents during induction of anesthesia (Table 2).

**Table 2:** Assessment of knowledge on cricoid pressure application in anesthetists working in Ayder comprehensive specialized hospital April 3–May 3, 2019

Knowledge on cricoid pressure	Number of anesthetists	Percentage
Where does cricoid cartilage lie		
In front of the thyroid cartilage	2	6.7
Behind the thyroid cartilage	2	6.7
Below the thyroid cartilage	25	83.3
Behind the esophagus	–	–
Why cricoid pressure is used		
Prevent aspiration of stomach contents during induction of anesthesia	30	100
Prevent patient breathing during anesthesia	–	–
Prevent patient vomiting during anesthesia	–	–
Prevent gastric gas insufflation during bag mask ventilation	–	–
The correct cricoid pressure in awake patient is		
10	15	50
20	4	13
30	–	–
50	–	–
Do not know	11	36.7
The correct cricoid pressure in anesthetized patient is		
10	1	3.3
20	8	26.7
30	20	66.7
50	–	–
Do not know	1	3.3
Correct measure if a patient vomits/regurgitate during cricoid pressure application		
Maintain the same force and suction the patient’s pharynx	3	10
Increase the force and suction the patient’s pharynx	9	30
Decrease the force and suction the pharynx	3	10
Release the force and suction the patient’s pharynx	15	50
When to release cricoid pressure		
After intubation	–	–
After inflation of the cuff	4	13
After confirmation the position of endotracheal tube	25	83.3
I don’t know	1	3.3

The correct response for force applied on the cricoid cartilage during rapid sequence induction in an awake and anesthetized patient was 50%. Thirty percent of the respondents believed increasing the cricoid pressure is the right measure if a patient vomits/regurgitates and only 50% replied releasing the force and suctioning the oropharynx is the correct measure (Table 2).

Majority, 90%, of the respondents do not mask ventilate during rapid sequence induction and they do aspirate the naso-gastric tube if present. Half, 43%, of the respondents have witnessed regurgitation during application of cricoid pressure and 93% do not remove the naso-gastric tube before rapid sequence induction. Seventy percent had experienced difficulty of endotracheal intubation during application of cricoid pressure (Table 3).

**Table 3:** Assessment of practice of cricoid pressure in anesthetists working in Ayder comprehensive specialized hospital April 3–May 3, 2019

Practice of cricoid pressure	Number of anesthetists	Percentage
Do you routinely mask ventilate during rapid sequence induction		
Yes	3	10
No	27	90
Do you aspirate before rapid sequence induction		
Yes	26	86.7
No	4	13.3
Do you remove NGT before rapid sequence induction		
Yes	2	6.7
No	28	93.3
Have you witnessed regurgitation during application of cricoid pressure		
Yes	13	43.3
No	17	56.7
Have you experienced difficulty to intubate during application of cricoid pressure		
Yes	21	70
No	9	30
Who applies cricoid pressure when you intubate		
Anesthetist	15	50
Anesthesia assistant	8	26.7
Nurses	5	16.7
Others	2	6.7

### Discussion

Most studies on practices of anesthesiologists regarding cricoid pressure application show a uniform poor theoretical knowledge among all categories of people and unacceptable variation in performance of the maneuver, which often leaves the patient at risk [6, 7].

This survey was conducted to determine the practice of application of cricoid pressure here in ACSH. Very few teaching institutes in our country have models where cricoid pressure application can be practiced to indicate forces that are to be used and those centers do not conduct a practical assessment of performance of CP by anesthetists.

Ninety-three (93) percent of the anesthetists had learned how to apply cricoid pressure on a patient during clinical practice or student attachments which shows very high value than a study done in New Zealand which shows only 53% were taught on a real patient. In this same study, around 20% had been taught on a model or mannequin whereas our study shows no one had that experience [8]. The reason for this could be that most of anesthesia schools in Ethiopia do not teach their students in a skills lab about appropriate application of cricoid pressure. As the respondents are graduates from different anesthesia schools in Ethiopia, it tells us that our teaching methods should include skills lab teaching of cricoid pressure application in a model (mannequin).

For the knowledge questions where the anatomic position of cricoid cartilage is and the use, the findings were 83% and 100% respectively. The same result was observed in the study done in New Zealand. This could be because this hospital is a teaching hospital and most of them are instructors. In this study only 67% of them had correctly answered the correct pressure applied on the cricoid cartilage which is slightly higher result than the study done by Korula G which was 27% [9]. This discrepancy could be because of the small sample we have used and the result could be falsely inflated.

In 1983 a study conducted on operating theatre personnel in UK and the USA showed that 70% had experienced a problem with the application of cricoid pressure which exposed the patient to risks of regurgitation. Ten percent had witnessed regurgitation in that group [7]. This contrasts with our finding where 57% had experienced regurgitation during rapid sequence induction. The reason for this staggering result could be explained from many perspectives. Many, 33%, did not identify the correct forces used in the application of cricoid pressure. Problems associated with distorted airway i.e. difficult laryngoscope placement, pharyngeal compression, and laryngeal distortion. It has been seen that incremental cricoid forces when applied on awake subjects lead to difficulty in breathing in half of them [10].

Endoscopic studies assessing the effect of cricoid pressure on the cricoid cartilage and vocal cords show that at forces of up to 44 N cause difficulty in ventilation was present in 50% of subjects and vocal cord closure occurred in 60%. Failure of ventilation was lower at 20 N than at 44 N [11]. This explains for the reason why 70% of the participants in this study had experienced difficulty of intubation which again is a higher result than the study done in India which was 55% [9]. This could be because in our study only 76% of the participants had anesthetist or anesthesia assistants to apply cricoid pressure for them, however, in the previous study it was around 90%.

Training makes a difference in the proper application of cricoid pressure in full stomach patients. A single training session using mannequins has shown to cause marked improvement in performance [5]. The use of simple instructions in an understandable form about the required force and use of simulators for practical training improves performance further [12, 13]. In addition, inexpensive and simple technique like using 50 ml syringe and depressing the plunger is reliable and linearly related to the force applied between 20 N and 50 N as explained by Flucker et al. [14].

### Conclusion

From this study we can learn that significant number of anesthetists had less knowledge of the correct force needed to apply on the cricoid cartilage, witnessed regurgitation of stomach content and majority had experienced difficulty of intubation while cricoid pressure was maintained. Use of simple model for training in higher academic institutions or training in refresher courses for individuals participating in cricoid pressure application in addition to theoretical base may improve the quality of good performance of cricoid pressure application.

## Limitations

To better identify weather the anesthetist’s new anatomy of cricoid cartilage and apply the appropriate force, it would have been better if we had had used anatomical model which shows the exact practice. Also, we had small sample size which makes it difficult to generalize to the whole anesthetist working in Ethiopia.

## Data Availability

The data sets generated during and/or analyzed during the current study are not publicly available but are available from the corresponding author on reasonable request.

## Abbreviations

ACSH: Ayder comprehensive specialized hospital
BSc: Bachelor of Science
CP: cricoid pressure
MSc: Master of Science
UK: United Kingdom
USA: United States of America
